# Comparative Gill Transcriptomics Reveals Unresolved Inflammation Under Chronic Hypoxia and Molecular Plasticity During Cyclic Hypoxia in *Salmo salar*

**DOI:** 10.3390/ani16132024

**Published:** 2026-07-02

**Authors:** Nicolás Salinas-Parra, Yannick Pombett, Felipe Stambuk, Matías Ilufi, Felipe Ramírez-Cepeda, Cristian A. Valenzuela, Carlos Soto, José Gallardo-Matus, Luis Mercado

**Affiliations:** 1 Grupo de Marcadores Inmunológicos en Organismos Acuáticos, Laboratorio de Genética e Inmunología Molecular, Instituto de Biología, Pontificia Universidad Católica de Valparaíso, Valparaíso 2373223, Chile; nicolas.salinas@pucv.cl (N.S.-P.); yannick.pombett@pucv.cl (Y.P.); felipe.stambuk@pucv.cl (F.S.); matias.ilufi@pucv.cl (M.I.); felipe.ramirez@pucv.cl (F.R.-C.); cristian.valenzuela@pucv.cl (C.A.V.); 2 Programa de Doctorado en Biotecnología, Pontificia Universidad Católica de Valparaíso, Valparaíso 2373223, Chile; 3 Salmones Camancha, Puerto Montt 5503642, Chile; csotov@camanchaca.cl; 4 Laboratorio de Genética y Genómica Aplicada, Escuela de Ciencias Del Mar, Pontificia Universidad Católica de Valparaíso, Avenida Universidad 330, Valparaíso 2373223, Chile; jose.gallardo@pucv.cl

**Keywords:** *Salmo salar*, chronic hypoxia, cyclic hypoxia, gills, stress, transcriptomics, innate immunity

## Abstract

Global aquaculture faces increasing threats from declining oxygen levels, with several key aquaculture regions now classified as hypoxic. The gills, responsible for oxygen uptake, pathogen-sensing, and immune responses, are the primary interface for this stress. However, the comparative effects of different hypoxic regimes on the gill resilience of Atlantic salmon (*Salmo salar*) remain poorly understood. In this study, Atlantic salmon were exposed during a 7-day trial to two different moderate hypoxic stress models, chronic hypoxia and cyclic hypoxia. A whole-genome oligo-microarray revealed that both hypoxic stress regimes cause transcriptomic shifts, driven in the chronic hypoxia group by upregulation of PRRs and other immune components and downregulation of DNA repair and cell cycle maintenance. On the other hand, the cyclic hypoxic group focused on physiological plasticity and rapid cellular adjustments. Additionally, RT-qPCR was used to evaluate key gene expression of hypoxia and immune response. The chronic hypoxia group was characterized by downregulation of *hif2a*, *gshpx*, and *gr-anx1* signaling, with a continuous upregulation state of *tnfa2*, suggesting an unresolved inflammatory state, often associated with apoptosis and gill damage. In contrast, the cyclic hypoxia group displayed an increased *epo* expression and recovery of antioxidant enzymes (e.g., *gsphx*), accompanied by normalized levels of *tnfa2* at the end of the 7-day trial. Therefore, chronic hypoxic stress imposes a significantly higher burden on the gills of Atlantic salmon than cyclic hypoxic stress, compromising their ability to respond to secondary stressors and impairing immune homeostasis.

## Simple Summary

Global aquaculture faces increasing threats from declining oxygen levels, with several key aquaculture regions now classified as hypoxic. However, the comparative effects of different hypoxic regimes on the gill resilience of Atlantic salmon (*Salmo salar*) remain poorly understood. In this study, Atlantic salmon were exposed during a 7-day trial to two different moderate hypoxic stress models, chronic hypoxia and cyclic hypoxia. A whole-genome oligo-microarray revealed that both hypoxic stress regimes cause transcriptomic shifts, driven in the chronic hypoxia group by upregulation of PRRs and other immune components and downregulation of DNA repair and cell cycle maintenance. On the other hand, the cyclic hypoxic group focused on physiological plasticity and rapid cellular adjustments. The chronic hypoxia group was characterized by downregulation of key hypoxia stress response markers, indicating exhaustion. In contrast, the cyclic hypoxia group displayed increased or recovered key hypoxia stress response markers. Therefore, chronic hypoxic stress imposes a significantly higher burden on the gills of Atlantic salmon than cyclic hypoxic stress, compromising their ability to respond to secondary stressors and impairing immune homeostasis.

## 1. Introduction

Oxygen homeostasis is the capacity of organisms to ensure that the supply and demand of oxygen are balanced [1]. When the oxygen levels are adequate to the metabolic requirements of a determined organism, they are in a normoxic state [1]. However, when the uptake of oxygen is below the threshold demand, the organism enters a hypoxic state [1,2]. Depending on the severity and time under hypoxic stress, it can induce morphological, physiological, biochemical, and behavioral responses, and in extreme cases, death [3,4,5,6]. Aerobic aquatic organisms, such as fish, are dependent on dissolved oxygen in their environment to support their metabolic activities. Due to global warming, human direct or indirect intervention, and eutrophication, ocean oxygen levels are depleting unevenly, with several key aquaculture regions now depicted as hypoxic areas, where oxygen uptake falls below the threshold required for normoxic maintenance [7].

Aquaculture is a global market that is rapidly growing [8], comprising fish, algae, shellfish, and plant farming. Animal aquaculture corresponds to 50.9% of the global aquaculture production, which in 2022 accounted for 94.4 million tons [9]. Atlantic salmon (*Salmo salar*) is the second most valuable species in global aquaculture, with 2.71 million tons produced in 2023 [10,11]. Moreover, worldwide aquaculture activities contribute to Sustainable Development Goals (SDGs) such as poverty reduction and zero hunger, among others [12]. Despite its economic importance, global aquaculture is facing continuous and new emerging threats, including rising sea temperatures, novel pollutants (e.g., microplastics and agricultural pesticides), acidification of the ocean, loss of oxygen in the ocean, and emerging pathogens [7,13,14,15,16,17]. As many of these threats are intertwined and have a strong and explored relationship with the immune system, the link between hypoxia and immune homeostasis is still not well understood. In this context, Atlantic salmon has demonstrated adaptability to declining oxygen levels in seawater farming cages. However, this adaptation is accompanied by chronic stress, reduced fish growth, loss of appetite, and a direct impact on fish welfare [17,18,19].

The gills, as a multifunctional organ, are responsible for gas exchange (e.g., oxygen uptake), oxygen chemoreception, and a physical and immunological barrier against pollutants and pathogens, respectively [20,21,22]. Therefore, the gills are the first responders to hypoxic stress, triggering signals that can lead to metabolic disorders, impaired antioxidant activity, morphological changes, and apoptosis [4,23]. On the other hand, the gills or gill cell-lines have been demonstrated to activate immune responses and pathogen sensing, while at the same time being an entry point to pathogens [24,25,26,27]. Nonetheless, the link between hypoxia and immunity (e.g., cytokines, PRRs) in gills remains underexplored.

Current research on fish hypoxia is highly fragmented across different experimental models, which can be classified by (i) intensity, ranging from lethal or sub-lethal low dissolved oxygen concentrations to mild or low hypoxic conditions [22,28], (ii) temporal, separated in chronic stress, one time-event, or cyclic hypoxia with re-oxygenation events [22,29], (iii) onset, which comprises a progressive or gradual hypoxic stress and acute or sudden hypoxia [23,30], (iv) and mixed models, which includes the addition of other stressors like temperature or pathogens [4,31]. The fact that different hypoxia models may or may not overlap complicates the comparison of results and their extrapolation to real-world production environments, where oxygen levels often fluctuate daily. Consequently, it remains unknown how mechanisms of molecular adaptation and immune resilience directly diverge when the same cohort of fish is subjected to sustained chronic stress versus dynamic cyclic regimes, leaving a gap in the understanding of which hypoxic regime presents a higher threat.

Given the biological complexity and the industry-wide impact of fluctuating oxygen levels, there is a critical need to determine which hypoxic regime poses a greater threat to salmonid health. Therefore, this study aims to provide a comprehensive comparative analysis of the effects of moderate chronic hypoxia and moderate cyclic hypoxia on the Atlantic salmon gill transcriptome. Our working hypothesis is that moderate chronic and cyclic hypoxia activate distinct, non-overlapping transcriptomic pathways, as a continuous oxygen deficit requires sustained systemic adjustments, whereas fluctuating levels demand recurrent molecular plasticity. Ultimately, this research seeks to elucidate how these stressors reprogram the immune system, offering new insights into the physiological limits and resilience of Atlantic salmon in intensive production environments.

## 2. Materials and Methods

### 2.1. Fish and Hypoxia Challenges

Post-smolts of Atlantic salmon Lochy strain from Camanchaca S.A. (Contao, Los Lagos, Chile), with a mean weight of (136.1 ± 26.8 g) and mean length of 21.6 cm, were acclimatized in a 0.75 m^3^ recirculation system for two weeks, with 24h light regime and 10 °C. Fish were fed daily with a commercial diet (New Power^®^, BioMar, Puerto Montt, Chile) at 1% of body weight. For the experiments, the feeding was stopped two days prior to the hypoxia challenge to standardize the baseline metabolic rate of the fish and ensure that the subsequent physiological and transcriptomic responses were driven solely by the hypoxic stress [32].

**Figure 1:**
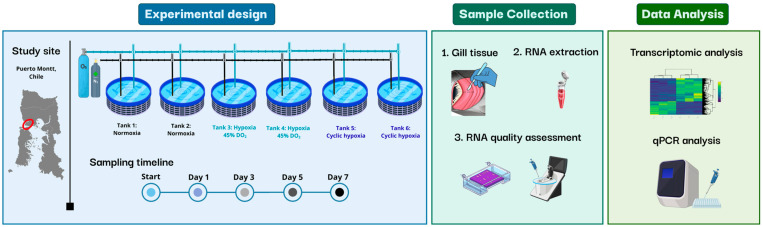
Experimental design of hypoxic challenges, sampling days and sample collection for subsequent processing.

For the hypoxia challenges, 120 fish were maintained in two replicate tanks per condition (60 fish each) in a density of 10 kg m^−3^ and exposed to two different hypoxia regimes: normoxia (90–100% dissolved oxygen saturation (DO)), continuous hypoxia (40–50% DO) and cyclic hypoxia (12 h normoxia followed by 12 hypoxia 50% DO) (Figure 1). The hypoxic treatment was performed by bubbling nitrogen gas into the tanks. Temperature and DO levels were monitored with a Polaric C dissolved oxygen meter (OxyGuard^®^, Farum, Denmark).

### 2.2. Sampling and RNA Extraction

Five fish in each experimental tank (yielding a combined sample of 10 fish per treatment group) were euthanized using an overdose of Benzocaine (Sigma-Aldrich, St. Louis, MO, USA) at 1, 3, 5, and 7 days after chronic or cyclic hypoxia. Thereafter, individuals were dissected, and gill samples were collected and stored in RNAlater^®^ (Invitrogen Corp., Carlsbad, CA, USA) (400 µL) at 4 °C for 8 days. Then, samples were kept at −80 °C until RNA extraction.

For RT-qPCR analyses, gill samples were organized as biological pools. At each sampling time and experimental condition, 10 fish were sampled, five from each of the two replicate tanks. Five biological pools were then generated per condition and sampling time, with each pool consisting of two fish, one from each tank. Therefore, the biological pool, rather than the individual fish, was considered the statistical unit for RT-qPCR analyses. As datasets were processed in independent RNA/cDNA synthesis and qPCR batches, hypoxia-induced transcriptional changes were analyzed relative to their corresponding batch-specific normoxia control. For graphical clarity, RT-qPCR figures display a single normoxic control bar.

Total RNA was extracted using TRIzol (Invitrogen Corp., Carlsbad, CA, USA) following the manufacturer’s instructions. All the samples were TRIzol-lysed using a tissue homogenizer (Fastprep-24, MP Biomedical, Solon, OH, USA) before total RNA extraction. To remove residual genomic DNA and enhance RNA quality, all samples were treated with DNAse at 1 Unit µL^−1^ (Thermo Fisher Scientific, Waltham, MA, USA) according to the manufacturer’s protocol. The purified RNA was quantified using a NanoDrop^®^ LITE spectrophotometer (Thermo Scientific, Waltham, MA, USA), and integrity was assessed using a 1% agarose gel electrophoresis. The RNA samples used in the microarray were sent to the USA, where the product was subjected to a rigorous quality control process before processing. For qPCR analysis, the samples showed high integrity and purity (i.e., A260/230 > 2.0 and A260/280 ratios > 1.8).

### 2.3. Microarray Analysis

A custom Atlantic salmon gene expression microarray (S.salar1; Thermo Fisher Scientific, Waltham, MA, USA) was designed using the latest publicly available mapped transcripts (GTF) and corresponding genome assembly Ssal_v3.1 (GCF_905237065.1). Briefly, transcript models were quality filtered (including removal of very small introns < 20 bp) and consolidated into “transcript clusters” (TCs) that represent gene-level models; potential chimeric clusters arising from overlapping loci were screened and resolved by removing bridging transcripts or splitting affected clusters. Redundant TCs were then collapsed using exact and near-identical sequence/probeset criteria, yielding 54,777 unique TCs from an initial ~64.8k TC set. Probe selection regions (PSRs) were derived from exon–intron structures, and 25-mer expression probes were scored and selected based on genomic/transcriptomic homology, sequence complexity (PolyNuc, DUST), and GC content; ~10 high-performing probes were assigned per TC with an effort to distribute probes across large PSRs. The final layout comprised ~566,270 total features, including 547,770 expression features (54,777 TCs × 10 probes), 17,000 antigenomic background controls, and 1500 hybridization/target-preparation controls, manufactured as a 96-array plate format with 6 µm features. RNA-sequencing data generated from samples were processed using TAC v4.0.2. Differential expression was estimated from gene count data using the R/Bioconductor package limma (v3.24) (implemented in TAC v4.0.2). Transcripts with an absolute fold change (FC) > 2 and a false discovery rate (FDR)–adjusted *p*-value (Padj) < 0.01 were considered differentially expressed (DE) and were used for functional enrichment analyses. Based on these results, DE transcripts were separated for each sampling day into upregulated and downregulated sets, which were then analyzed as follows: pathway enrichment analyses for the different transcript lists were performed using the ClueGO [33] and CluePedia [34] plugins in Cytoscape (v3.10.3). Kyoto Encyclopedia of Genes and Genomes (KEGG) pathway analysis was used to identify key signaling pathways containing DE mRNAs. Gene Ontology (GO) analysis was conducted to explore the biological roles of DE mRNAs across the Molecular Function (MF), Biological Process (BP), and Cellular Component (CC) categories. Enrichment analyses (right-sided hypergeometric test) were performed using the GO database, and *p*-values were adjusted using the Benjamini–Hochberg method (*p* < 0.05). Based on these results, we selected up to 10 GO terms with the lowest *p*-values from upregulated and downregulated gene sets across all categories, and graphs were generated using SRplot https://www.bioinformatics.com.cn/en (accessed on 5 January 2026).

### 2.4. Protein–Protein Interaction Analysis

To examine the relationships and functions of the DE mRNAs, a protein–protein interaction (PPI) analysis was performed using the STRING database https://string-db.org/ (accessed on 15 September 2025). The upregulated and downregulated mRNAs were visualized using Cytoscape (v3.10.3) with high confidence ≥ 0.5 and hiding disconnected nodes in the network. The significant modules in the network were scored > 9 using the MCODE (Molecular Complex Detection) [35] Cytoscape plugin.

### 2.5. qPCR

Approximately 1000 ng of the extracted RNA was reverse transcribed to cDNA using the RevertAid First strand cDNA Synthesis kit (Thermo Scientific, Waltham, MA, USA) according to the manufacturer’s instructions. Quantitative PCR (qPCR) was performed using 2xPCR master mix (Kapa Biosystems, Wilmington, MA, USA) and the AriaMx qPCR System (Agilent, Santa Clara, CA, USA). The real-time analysis program consisted of 1 cycle of 95 °C for 3 min and 40 cycles of 95 °C for 5 s and 60 °C for 30 s, with fluorescence detection at the end of each 60 °C step. Elongation factor 1α (*elf-1α*), glyceraldehyde-3-phosphate-dehydrogenase (*gapdh*), and hypoxanthine-guanine phosphoribosyltransferase (*hprt*) were used as reference genes [35,36,37,38]. The expression of target genes (Table 1) was analyzed using the 2^−ΔΔCt^ method [39,40]. The validation of the microarray through RT-qPCR can be found on Figure S1.

### 2.6. Statistical Analysis

Normality distribution test was assessed by the Shapiro–Wilk test. To identify and remove outliers, the Rout method was performed. Significant differences were evaluated by a two-way Analysis of Variance (ANOVA), considering hypoxic regimes (chronic and cyclic) and exposure time points (D1, D3, D5, and D7) as the independent factors. To determine differences between specific experimental groups and analyze temporal kinetics, Tukey’s honest significant difference (HSD) multiple comparisons test was applied as a post hoc analysis. Graphical representations of the results were made using GraphPad Prism 10.1.1. and the fold changes in genes were presented as mean ± standard error of the mean (SEM). Differences were considered significant when *p* was < 0.05.

## 3. Results

### 3.1. Whole-Genome Oligo-Microarray

#### 3.1.1. Chronic Hypoxia

**Figure 2:**
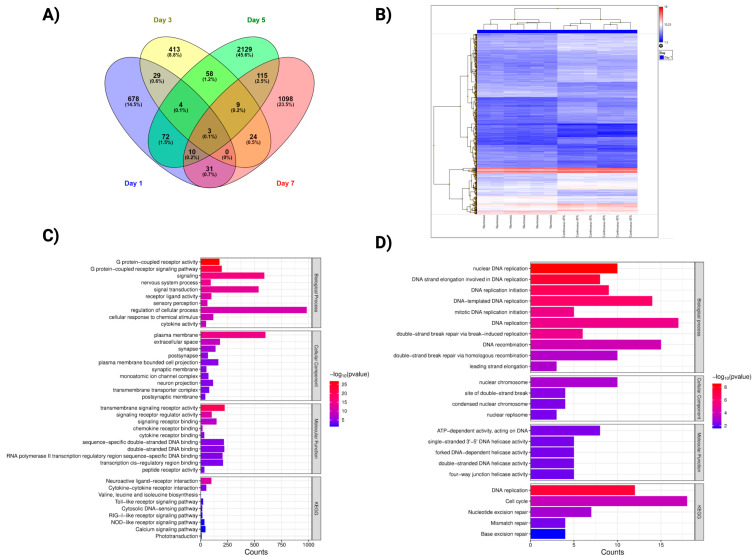
Chronic hypoxia (45%)-induced gene expression changes assessed by microarray. (**A**) Venn diagram of differentially expressed genes (DEGs) in the gill of *Salmo salar* under hypoxia condition. (**B**) Hierarchical clustering of gene expression profiles in Atlantic salmon gills under normoxic and chronic hypoxia (45%) conditions. The heatmap represents normalized transcript levels (log_2_ fold change), with hierarchical clustering performed using Euclidean distance and the complete linkage method. Genes and samples are grouped according to their expression similarity. Distinct clusters highlight differential gene expression patterns associated with hypoxic exposure. Gene ontology and Kyoto Encyclopedia of Genes and Genomes enrichment analysis of upregulated (**C**) and downregulated (**D**) genes over experiment.

To evaluate the transcriptomic effect of chronic hypoxia stress on the gill of Atlantic salmon, a Venn diagram for each measured time point was generated (Figure 2A). A total of 4672 DEGs were identified over the 7 days, with the majority of unique DEGs occurring at D5 (45.6%) and D7 (23.5%). Consistently modulated genes identified from D1 to D7 were three (0.1%), while 31 (0.7%) genes were shared between D1 and D7. The hierarchical clustering (Figure 2B) revealed a clear visual separation between the normoxia group and the chronic hypoxia group, with high similarity among biological replicates. The KEGG enrichment of upregulated DEGs (Figure 2C) highlighted a strong activation of innate immune sensing pathways, including NOD-like receptor, Toll-like receptor, RIG-I-like receptor, alongside neuroactive ligand–receptor interaction, and metabolic pathways. On the other hand, downregulated DEGs (Figure 2D) were associated with DNA maintenance and proliferation, such as DNA replication, cell cycle, nucleotide excision repair, mismatch repair, and base excision repair. The upregulation of mucin 2-like was the highest at D1, followed by myelin and lymphocyte protein-like at D3, eosinophil peroxidase-like at D5, and C-C motif chemokine 20 on D7. On the other hand, the most downregulated genes were NLR family CARD domain-containing protein 3-like at D1, proto-oncogene c-Fos at D3, myosin heavy chain, fast skeletal muscle-like at D5, and THO complex subunit 3-like at D7. A detailed summary of the highest upregulated and downregulated DEGs for each time point of chronic hypoxic stress can be found in Table 2 and Table 3, respectively.

#### 3.1.2. Cyclic Hypoxia

**Figure 3:**
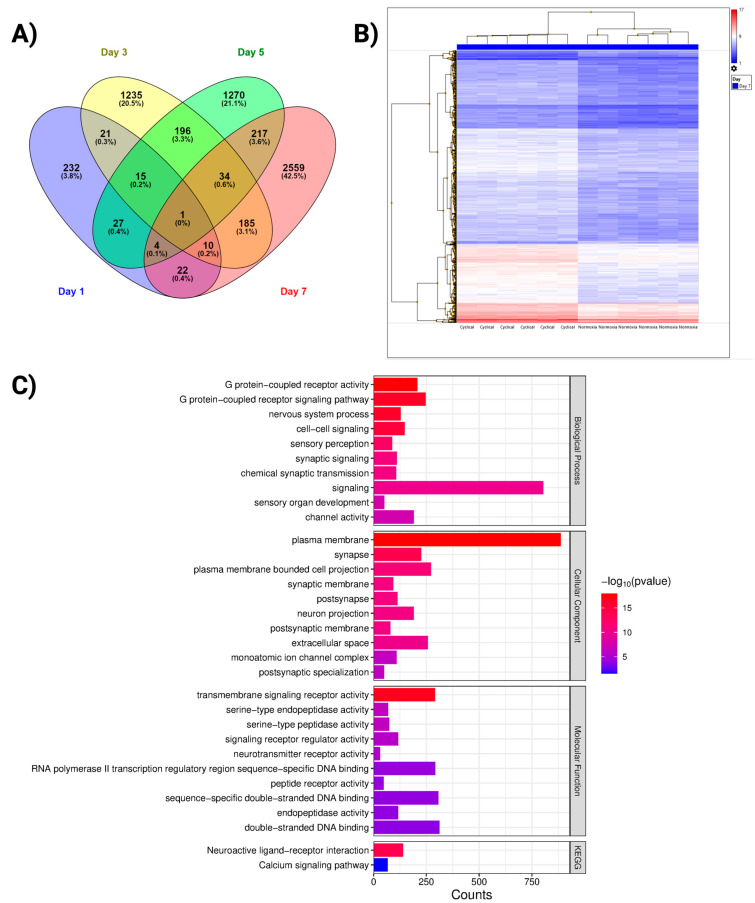
Cyclic hypoxia-induced gene expression changes assessed by microarray. (**A**) Venn diagram of differentially expressed genes (DEGs) in the gill of *Salmo salar* under hypoxia condition. (**B**) Hierarchical clustering of gene expression profiles in Atlantic salmon gills under normoxic and cyclic hypoxia conditions. The heatmap represents normalized transcript levels (log_2_ fold change), with hierarchical clustering performed using Euclidean distance and the complete linkage method. Genes and samples are grouped according to their expression similarity. Distinct clusters highlight differential gene expression patterns associated with hypoxic exposure. Gene ontology and Kyoto Encyclopedia of Genes and Genomes enrichment analysis of upregulated (**C**) genes over the experiment.

Exposure to cyclic hypoxia resulted in 5314 DEGs (Figure 3A), with the peak of unique DEGs identified at D7 (42.5%). Overlapped DEGs were lower than unique DEGs, similar to chronic hypoxia stress, with just 22 (0.4%) conserved in D1 and D7, while only one DEG was shared across D1 to D7. Mirroring the chronic hypoxia group, hierarchical clustering displayed distinct separation between normoxia and cyclic hypoxia treatments (Figure 3B). However, unlike the chronic hypoxia group, KEGG analysis of upregulated genes showed enrichment primarily in neuroactive ligand–receptor interaction and calcium signaling pathways, with no significant enrichment of direct immune-related pathways. At the individual gene level, alpha-1-antitrypsin homolog, hemopexin a, APA11, apoa1 apolipoprotein A-I, and 60S ribosomal protein L18a were the most upregulated genes at D1, D3, D5 and D7, respectively. On the contrary, the most downregulated genes were uncharacterized LOC106594338 at D1, proto-oncogene c-Fos at D3, cilia- and flagella-associated protein 251-like at D5, and zinc finger protein 501-like at D7. The most modulated genes for each time point under cyclic hypoxic stress are summarized in Table 4 (upregulated genes) and Table 5 (downregulated genes).

### 3.2. Expression of Angiogenic and Oxygen-Sensing Markers

**Figure 4:**
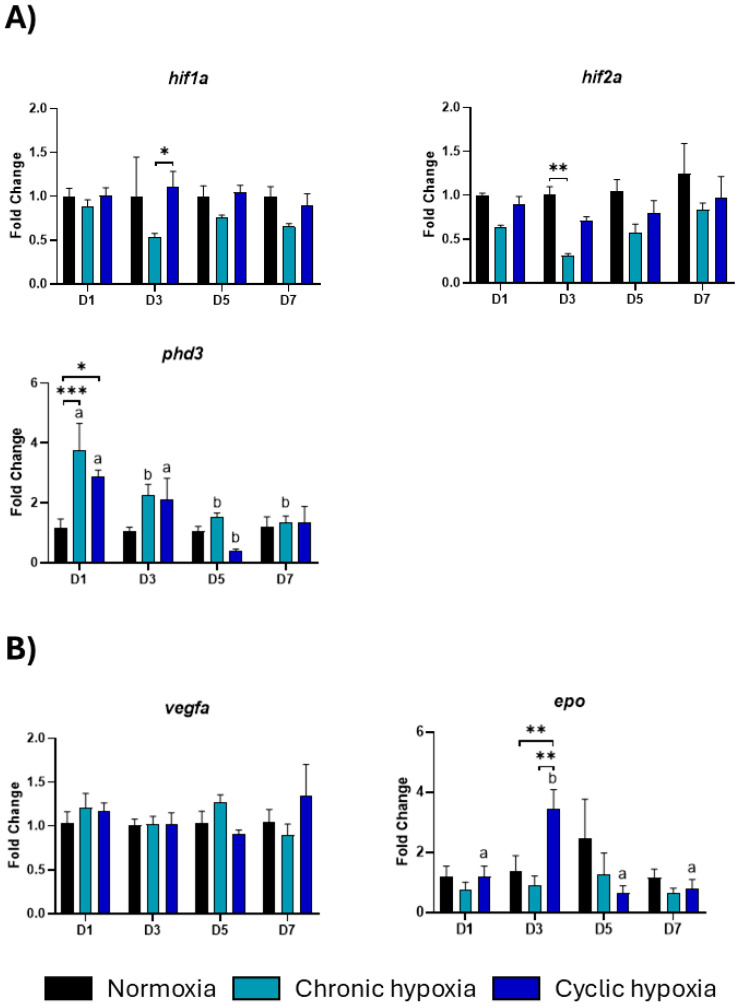
Expression profiles of hypoxia and circulatory markers in Atlantic salmon gills under chronic and cyclic hypoxia. Gene expression levels are expressed as fold changes relative to the normoxic control groups. (**A**) Relative expression of hypoxia-inducible factor (*hif1a*, *hif2a*, and *phd3*) genes. (**B**) Relative expression of genes associated with blood circulation and vascular regulation (*vegfa* and *epo*). Data are presented as mean ± standard error of the mean (SEM) (*n* = 5 pools of two fish per treatment). Statistically significant differences between treatments within the same sampling day are indicated with a mark (*, *p* < 0.05), two marks (**, *p* < 0.01), or three marks (***, *p* < 0.001). Different lowercase letters denote significant differences (*p* < 0.05) between time points within the same treatment group.

The relative expression of *hif1a* (Figure 4A) was lower in the chronic hypoxia group than the cyclic hypoxia group (D3), while no significant differences were found in either group compared to normoxia. On the other hand, *hif2a* was downregulated in the chronic hypoxia (D3) group in relation to the normoxia group.

The mRNA levels of the *phd3* gene (Figure 4A) increased in chronic hypoxia and cyclic hypoxia groups (D1) in comparison to the normoxia group, followed by a downregulation observed in the chronic (D3, D5, and D7) and cyclic hypoxia (D5) groups in relation to D1.

Regarding the circulatory system markers, the *vegfa* expression (Figure 4B) displayed no significant differences across the experiment. Finally, the *epo* (Figure 4B) gene exhibited an upregulation in cyclic hypoxia (D3) regarding the chronic hypoxia and normoxia groups, being significantly higher than all other measured time points.

### 3.3. Oxidative Stress

**Figure 5:**
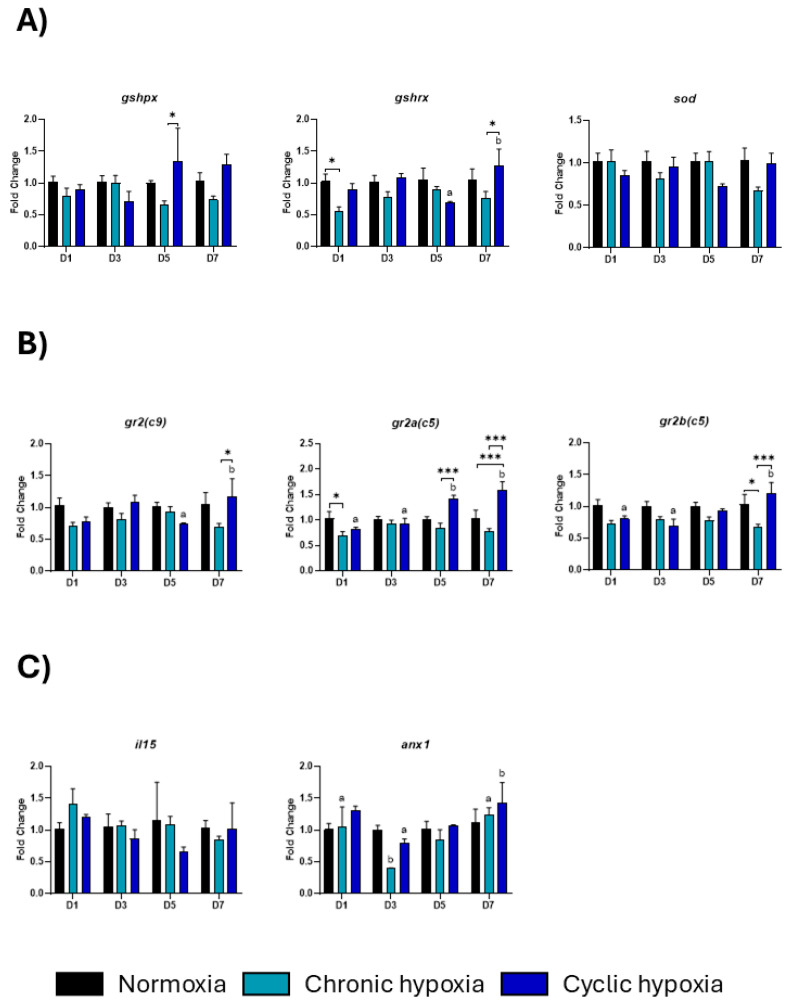
Relative expression of genes associated with oxidative stress, cortisol-responsive signaling receptors, and regulatory immune signaling markers in the gills of Atlantic salmon exposed to chronic and cyclic hypoxia. Gene expression levels are presented as fold changes relative to normoxic controls. (**A**) Relative expression of oxidative stress genes (*gshpx*, *gshrx*, and *sod*), (**B**) cortisol-responsive signaling receptors (*gr2(c9)*, *gr2a(c5)*, and *gr2b(c5)*), and (**C**) regulatory immune signaling markers (*anx1* and *il15*). Data are presented as mean ± standard error of the mean (SEM) (*n* = 5 pools of two fish per treatment). Statistically significant differences between treatments within the same sampling day are indicated with a mark (*, *p* < 0.05), or three marks (***, *p* < 0.001). Different lowercase letters denote significant differences (*p* < 0.05) between time points within the same treatment group.

The relative expression of *gshpx* (Figure 5A) was upregulated in the cyclic hypoxia group compared to the chronic hypoxia group (D5). Similarly, the *gshrx* gene (Figure 5A) was downregulated in the chronic hypoxia group (D1) regarding the normoxia group, while its expression was increased in the cyclic hypoxia group in comparison to the chronic hypoxia group (D7). Additionally, the levels of *gshrx* in the cyclic hypoxia group fluctuated from D1 to D7, with no significant increases or decreases compared to the normoxia group. The expression of *sod* exhibited a similar pattern to *gshrx*, with non-significant fluctuations from D1 to D7 in both hypoxia groups.

### 3.4. Stress Response

Transcriptional levels of *gr2(c9)* (Figure 5B) were significantly increased in the cyclic hypoxia group from D5 to D7, marked with a significant upregulation relative to the chronic hypoxia group (D7). Similarly, the *gr2a(c5)* (Figure 5B) was upregulated in the cyclic hypoxia group compared to the normoxia (D7) and the chronic hypoxia groups (D5 and D7) while the chronic hypoxia group was downregulated in comparison to the normoxia group (D1)). In the chronic hypoxia group, *gr2b(c5)* (Figure 5B) was downregulated (D7) regarding the normoxia and cyclic hypoxia groups (D7). Finally, all three genes (Figure 5B) increased their expression towards D5 or D7 in the cyclic hypoxia groups compared to chronic hypoxia groups.

The expression of *il15* displayed no significant differences in both hypoxia groups, while *anx1* followed a temporal pattern, characterized by an initial reduction at D3 and a progressive increase towards D7 in both hypoxia groups.

### 3.5. Cytokines and Pathogen-Recognition Receptors

**Figure 6:**
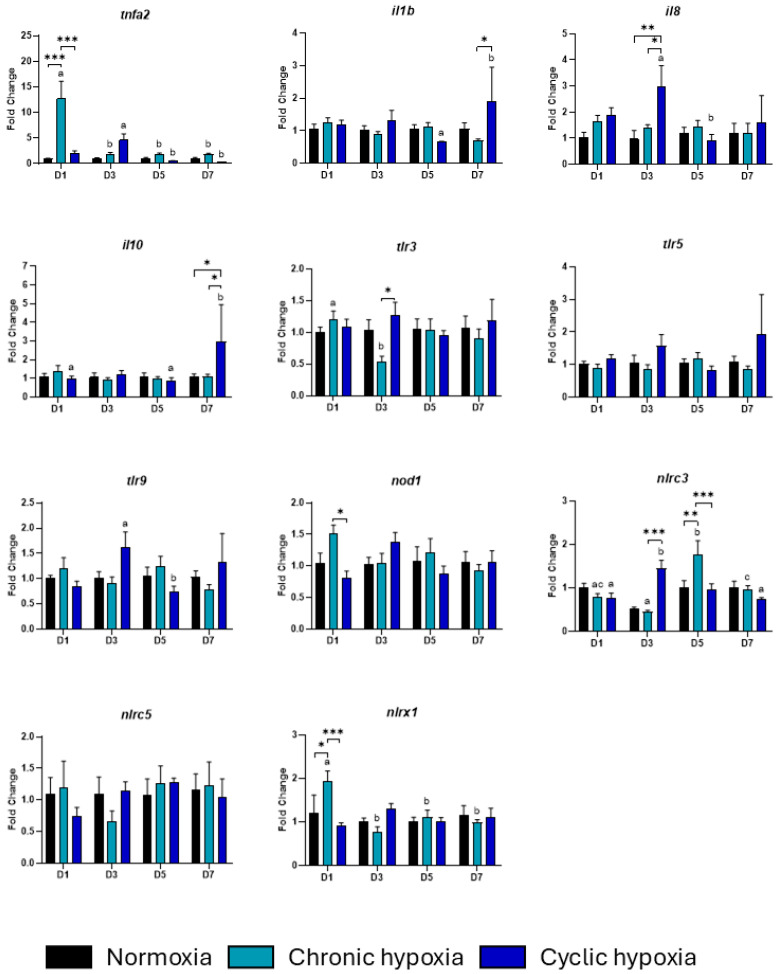
Immunomodulatory effects of chronic and cyclic hypoxia in gills of Atlantic salmon. Gene expression levels are presented as fold changes relative to normoxic controls (*tnfa2*, *il1b*, *il8*, *tlr3*, *tlr5*, *tlr9*, *nod1*, *nlrc3*, *nlrc5*, and *nlrx1*). Data are presented as mean ± standard error of the mean (SEM) (*n* = 5 pools of two fish per treatment). Statistically significant differences between treatments within the same sampling day are indicated with a mark (*, *p* < 0.05), two marks (**, *p* < 0.01), or three marks (***, *p* < 0.001). Different lowercase letters denote significant differences (*p* < 0.05) between time points within the same treatment group.

To evaluate the comparative effects of chronic and cyclic hypoxia on innate immunity, the expression of key immune markers was assayed (Figure 6). The relative expression of *tnfa2* was upregulated in the chronic hypoxia group compared to the normoxia and cyclic hypoxia groups (D1), followed by a significant decrease maintained from D3 to D7. Similarly, *tnfa2* transcriptomic levels increased in the cyclic hypoxia group (D3), followed by a downregulation in D5 and D7. The expression of *il1b* increased in the cyclic hypoxia group from D5 to D7, displaying a significant difference compared to chronic hypoxia (D7). The *il8* gene expression was upregulated in both hypoxia groups in comparison to normoxia (D3), followed by a downregulation in the cyclic hypoxia group from D3 to D5. On the other hand, the anti-inflammatory cytokine gene *il10* displayed an increased expression in the cyclic hypoxia group regarding normoxia and chronic hypoxia groups (D7) and within the same group on D1 and D5.

Among the pathogen-recognition receptors (PRRs) evaluated (Figure 6), *tlr3* was downregulated in the chronic hypoxia group (D3) compared to the cyclic hypoxia group, with a marked decrease from D1 to D3. The transcript levels of *nlrc3* increased in the cyclic hypoxia group compared to the chronic hypoxia group (D3), displaying a significant fluctuating “decrease–increase” pattern from D1 to D7. Likewise, the expression of *nlrc3* in the chronic hypoxia group followed a similar trend from D1 to D7, reaching its maximum at D5, being significantly upregulated in comparison to cyclic hypoxia and normoxia groups (D5). Regarding *nod1* and *nlrx1*, a significant upregulation was displayed in the chronic hypoxia compared to cyclic hypoxia group (D1) and cyclic hypoxia and normoxia groups (D1), respectively. No significant differences were found between both hypoxic stress groups and the normoxia group on *tlr5*, *tlr9*, and *nlrc5*.

## 4. Discussion

The global aquaculture industry is constantly facing several pre-existing and emerging challenges [7,13,14,15,16]. The hypoxic stress in farmed fish has been demonstrated to cause oxidative stress, metabolic shifts, performance reduction, immune system impairment, and, in more severe cases, death [3,4,5,6]. However, several hypoxic stress models exist, exhibiting different responses, which can also vary across fish species [4,22,24,28,29,31]. Therefore, to comparatively understand the effect on the transcriptomic changes and immunity, this study compares mild chronic hypoxia and cyclic hypoxia in the gill of Atlantic salmon, a prominent global farmed species.

Both chronic hypoxia and cyclic hypoxia stress generated a transcriptomic shift in Atlantic salmon, with a total of 4672 and 5314 unique DEGs, respectively. As most of the DEGs expressed were unique DEGs and not consistently modulated across the 7-day trial, both hypoxic stresses caused a transcriptomic shift at each measured time point. Additionally, the modulation of genes in chronic hypoxia appears to be more extreme, as on D5 the number of unique DEGs increases dramatically (5-fold) compared to D3, which was reduced again to half on D7. Conversely, cyclic hypoxia stress displayed a similar increase at D3 (6-fold), which was stabilized until D7 (2-fold increase). In a farming sea-cage environment, fish can encounter longer exposure to hypoxic stress, which can also range from chronic to cyclic hypoxic stress [18,41,42]. Therefore, given the absence of a return to baseline levels or a plateau in consistently expressed DEGs, further studies exceeding 7 days are recommended to determine the long-term homeostatic state and ensure that results are transferable to industrial aquaculture environments.

The chronic group exhibited a sterile inflammatory modulation, as evidenced by the enrichment of PRR and cytokine signaling pathways. This sustained immune activation, coupled with the downregulation of high-energy processes like DNA repair and cell cycle maintenance, points to a classic metabolic–immune trade-off. By diverting ATP away from cellular growth to fuel a sterile inflammatory response, chronic hypoxia imposes a significant allostatic load on the host [43,44]. In contrast, the cyclic hypoxic stress appears to focus on physiological plasticity and rapid cellular adjustment rather than the broad immunological restructuring under constant hypoxic stress. The activation of calcium signaling in the cyclic group likely reflects the demands of increased branchial ventilation as a physiological compensation [45]. Consequently, while cyclic hypoxia appears to demand rapid physiological and calcium-mediated adjustments, the immunological restructuring under chronic stress may lead to a state of immune dysregulation, potentially leaving the host less resilient to pathogen exposure, as seen in other studies [32,46].

The hypoxia-inducible factor (HIF) is a master regulator transcription factor of hypoxia responses [47]. The HIF proteins are conserved across fish and mammals, including Atlantic salmon, and are constantly expressed in cells as an essential molecule for cell survival [47,48]. However, to avoid non-necessary low oxygen responses, the constant expressed HIFs are repressed by prolyl hydroxylases, encoded by the *phd3* gene, marking HIF proteins for degradation [49]. Under hypoxic stress, HIF transcriptional expression in teleosts has been observed as both rapid and late response [48,50,51,52], with divergences probably due to fish species and the severity of the hypoxic regime. In the present study, the absence of *hif1a* or *hif2a* upregulation at any measured time point in both hypoxia groups compared to the normoxia group, parallel to a significant early increase in *phd3* expression at D1, suggests a rapid autoregulatory feedback loop. Therefore, the initial hypoxia-sensing event likely occurred within the first hours of exposure. Furthermore, the localized downregulation of *hif1a* in the chronic group compared to the cyclic group at D3, along with the downregulation of *hif2a* under chronic hypoxia relative to normoxia at the same time point, indicates a transition from acute compensation to a state of transcriptional dampening.

The vascular endothelial growth factor A (VEGFA) is a key regulator of angiogenesis, affecting endothelial cell proliferation, migration of angioblasts, and differentiation, representing a biomarker of the vascular and hematopoietic system [53,54]. Herein, our results exhibited no differences in both hypoxia stress groups against the normoxia group. Similar to *hif* expression, *vegfa* expression is modulated as a rapid response within 24 h and is positively correlated to *hif* [55,56,57,58]. Therefore, the initial differential expression of *vegfa* likely occurred within the first hours of exposure.

As another component of the hematopoietic system, erythropoietin is a glycoprotein that regulates erythropoiesis and is encoded by the *epo* gene [59]. The modulated expression of *epo* is a common acclimatization response to hypoxia in teleost fish to maintain oxygen levels and delivery [59,60]. Likewise, when Atlantic salmon were exposed to cyclic hypoxic stress, *epo* was significantly upregulated. The absence of similar *epo* expression in the chronic group, despite the constant hypoxic stimulus, points toward transcriptional exhaustion or a metabolic prioritization that favors immediate survival. Consequently, cyclic hypoxia may promote a more resilient phenotype through periodic “resetting” of the oxygen-sensing machinery.

The antioxidant system comprises a wide range of molecules that maintain redox balance, protecting the cell against free radical species [61]. Among them are enzymes such as glutathione peroxidase (GSH-PX), glutathione reductase (GSH-RX), and superoxide dismutase (SOD), catalyzing the detoxification of H_2_O_2_ and organic hydroperoxides, reduction in oxidized glutathione, and dismutation of O_2_^−^, respectively [61]. Under chronic hypoxia, the early downregulation of *gshpx* suggests an impairment in maintaining the antioxidant defenses, as hypoxic conditions are characterized by the imbalanced production of ROS [5,62]. Conversely, the cyclic hypoxia group demonstrated a different recovery pattern, characterized by an upregulation of *gshpx* at D5 and *gshrx* at D7 compared to the chronic hypoxia group. This divergence could imply a prevention of antioxidant impairment due to the intermittent nature of the stimulus, allowing the antioxidant system to “reset” between cycles. Consequently, the chronic group may have experienced the accumulation of ROS, which serves as a physiological trigger for the sterile inflammation and DNA damage observed in the transcriptomic profile [63,64].

Glucocorticoid receptors (GRs) are stress response-related molecules that bind to glucocorticoids (e.g., cortisol), triggering an immunosuppressive response primarily by transactivating regulatory mediators such as annexin A1 (Anx1) [65,66,67]. Under cortisol presence in fish, if the stress response is not resolved, GR expression is reduced, the immune system is suppressed, and pro-apoptotic genes are upregulated [65,66,68]. In the present study, the downregulation of *gr2b(c5)* at D7 in the chronic hypoxia group revealed an impairment in the feedback mechanisms required to resolve inflammation, as seen in the upregulation of *tnfa2* at D1. On the contrary, the expression of *gr* paralogs increased towards D7 in the cyclic hypoxia group, likely allowing the regulation of the sterile inflammation described by the transcriptomic profile. Collectively, these findings suggest that prolonged chronic hypoxia stress may be more taxing and harmful to Atlantic salmon than cyclic hypoxia, as the constant stress signals and unresolved proinflammatory response (e.g., TNF-α) in gills have been demonstrated to cause structural damage and cell apoptosis [4].

The gills function as a physical barrier to environmental pathogens, but also as an entry point, being able to respond to pathogen stimuli through the release of proinflammatory cytokines to defend against them [20,24,69]. The data from this study revealed an early upregulation of *tnfa2* in the chronic hypoxia group, indicating sterile inflammation. While *tnfa2* is a known cytokine associated with pathogen defense, its presence under hypoxic stress is related to causing apoptosis and structural remodeling [32,70,71,72]. In contrast, the cyclic hypoxia group exhibited a lesser non-significant upregulation, suggesting a more controlled and less detrimental response.

Vertebrates, including teleosts, possess intracellular and extracellular PRRs to sense and respond to pathogens or danger signals, including families such as toll-like receptors (TLRs), RIG-I-like receptors (RLRs), and NOD-like receptors (NLRs) [73]. The early suppression of *tlr3* in the chronic hypoxia group, the gene encoding for Toll-like receptor 3, suggests a rapid sensing impairment or exhaustion of the innate immune detection machinery [73]. Furthermore, while both hypoxia stress groups displayed a late fluctuating *nlrc3*, the anti-inflammatory signaling PRR NLR family CARD domain containing 3 [74], the response was delayed in comparison to cyclic hypoxia.

Regarding the interleukin 1-beta (IL-1β) and interleukin 8 (IL-8), responsible for the pro-inflammatory response and recruitment of neutrophils, respectively [75], *il8* was upregulated in the cyclic hypoxia group regarding the normoxia group, with no significant differences found in *il1b* in both hypoxic stress groups compared to the normoxia group. Conversely, other studies have found an increased expression of *il1b* and *il8* after chronic hypoxic treatment [4,76,77,78], and no significant differences in cyclic hypoxia [79]. Similar to the *hif* expression, these differences may be attributed to fish species and the hypoxia stress regime.

Finally, in this study biological pooling was implemented for RT-qPCR analyses. While replicating experimental tanks is the gold standard in aquaculture research to account for potential tank-associated environmental variances, the strategy of pooling individuals across duplicate tanks aimed to reduce baseline noise, stabilize variance, and enhance the statistical power to identify genuine, treatment-specific differentially expressed genes under constrained sample sizes [80,81,82]. Nevertheless, this approach limits our capacity to statistically segregate true tank-level variance from global residual error. Therefore, future studies utilizing independent, non-pooled individuals across an expanded number of replicate systems are encouraged to further decouple individual biological plasticity from macro-environmental systems variance.

## 5. Conclusions

The present study demonstrated that while both chronic and cyclic hypoxia induce significant transcriptomic restructuring in the gills of Atlantic salmon, differences arise. The chronic hypoxic stress drives Atlantic salmon into a state of metabolic and immune impairment, characterized by the downregulation of DNA maintenance, oxygen-sensing machinery (*hif2a*), and antioxidant defense (*gshrx*). Additionally, the downregulation of specific glucocorticoid paralogs (*gr2b(c2)*) highlights a distinct modulation of stress response pathways under constant atmospheric conditions. In contrast, cyclic hypoxic stress appears to promote physiological plasticity. The periodic return to normoxia is likely acting as a “homeostatic reset”, allowing Atlantic salmon to activate compensatory responses, such as the upregulation of *epo*, *gshpx,* and *gshrx*. Additionally, the transient nature of *tnfa2* at D3, alongside a late progressive increase in *il1b* and *il10* expression towards D7 suggests that cyclic hypoxia maintains a structurally regulated and alert immune status.

In summary, chronic hypoxia imposes a significantly higher physiological burden that compromises the ability of the host to respond to secondary stressors. These data provide a biological foundation for oxygen management in aquaculture, indicating that mitigation efforts must focus on eliminating extended periods of continuous hypoxia. Therefore, providing brief periods of normative oxygenation can serve as a practical operational strategy to trigger compensatory responses and prevent gill exhaustion in the Atlantic salmon farming industry. Further long-term studies are required to determine if these conditions can be maintained beyond the 7-day window.

## Figures and Tables

**Table 1:** Primers used in RT-qPCR. F and R: Forward and reverse sequences, respectively. Tm: Melting temperature (°C). Primers efficiency (E). Accession number obtained from National Center of Biotechnology Information (NCBI).

Gene	Primers Sequence	E	Accession Number
*ef-1a (Elongation factor 1 alpha)*	F: GCTTACAAAATCGGCGGTAT R: CTTGACGGACACGTTCTTGA	2.02	NM_001141909.1
*hprt (hypoxanthine-guanidine phosphoribosyl transferase)*	F: CTGACATTGCCCCTCAGATT R: CACGCCTCAGAAATGTTCAA	1.99	XM_014212855.2
*gapdh (glyceraldehyde-3-phosphat dehydrogenase)*	F: CCTGCAGAAGGGAATCAAAGTCGT R: TCTCATGGGGCTTCAAACACT	1.99	NM_001123561.1
*hif1a (hypoxia inducible factor, isoform 1)*	F: AAGTTGTTTGCCGTCTGTCC R: CACCAAGTGTCTCGGTCTCT	1.99	NM_001140022.1
*hif2a (hypoxia inducible factor, isoform 2)*	F: AAGTTCACCTACTGCGACGA R: TACTGTCCACTCACTGCCTG	1.99	XM_045710198.1
*phd3 (prolyl hydroxylase, isoform 3)*	F: GTGCTATCCAGGAAATGGG R: TGCAACGTAGGATTTGCC	2.06	XM_014194431.2
*epo (erythropoietin)*	F: TGATGCTGTTGGAGTGGACC R: ACTGAACCTCCTGAGCTTGC	1.93	XM_014207097.2
*vegfa (vascular endothelial growth factor A)*	F: ACGGTTGGAAGTTTTGACGG R: AGTCTCCAAATGCGAGTCCA	2.08	XM_014144645.2
*gshpx (glutathione peroxidase)*	F: GATTCGTTCCAAACTTCCTGCTA R: GCTCCCAGAACAGCCTGTTG	1.98	XM_014213339.2
*gshrx (gluthatione reductase)*	F: CCGCGCCTCATATCCTCATT R: CTATGACGCTGCGCTTAGGA	1.98	XM_014199132.2
*sod (superoxide dismutase)*	F: CCACGTCCATGCCTTTGG R: TCAGCTGCTGCAGTCACGTT	2.05	NM_001123587.1
*gr2(c9) (glucocorticoid receptor 2, chromosome 9)*	F: CTGGAGAGAGAGATGCGTCAG R: GAAGCCCTCTGCCTTTCCTAA	2.07	XM_045724204.1
*gr2a(c5) (glucocorticoid receptor 2, isoform a chromosome 5)*	F: AGCCGTGGAAGGTACAGG R: GCCATGAGACACTTGCGGA	1.99	XM_014198677.2
*gr2b(c5) (glucocorticoid receptor 2, isoform b, chromosome 5)*	F: GAGAGCCGTGGAAGGGC R: CAGGTTCATCCCTGCCAT	1.9	XM_014198678.2
*il15 (interleukin 15)*	F: GGAGTTGGAGGTTGTCCTGA R: ATTGGCGTTCATGTTCTTCC	1.9	NM_001279065.1
*anx1 (annexin 1)*	F: ATGGTGAACCAGGAACTTGC R: ATAGCCTTTGCCACATCCAC	1.9	NM_001141271.1
*tnfa2 (tumor necrosis factor alpha, isoform 2)*	F: CTGTGTGGCGTTCTCTTAATAGCAGCTT R: CATTCCGTCCTGCATCGTTGC	1.9	NM_001124374.1
*il1b (interleukin 1 beta)*	F: GTCACATTGCCAACCTCATCATCG R: GTTGAGCAGGTCCTTGTCCTTGA	2.0	NM_001123582.1
*il8 (interleukin 8)*	F: TGTCGCTGCATTGAGACGGAAA R: AGCGCTGACATCCAGACAAATC	1.83	NM_001140710.3
*il10 (interleukin 10)*	F: ATGAGGCTAATGACGAGCTGGAGA R: GGTGTAGAATGCCTTCGTCCAACA	1.94	XM_045705802.1
*tlr3 (Toll-like receptor 3)*	F: CTGCGGTCCTGACTTTAATGGTGA R: AAGCTTCGTTCCACCCAGGTTT	1.9	XM_014195262.2
*tlr5 (Toll-like receptor 5)*	F: GCATGACTCAGTATGGCTTTG R: GTGGATTTTTGTCCCTCGGA	1.9	HQ664667.1
*tlr9 (Toll-like receptor 9)*	F: CCAGTTCCGTTGGCTGGAGG R: GACTGAGTAGAGAAGGATGG	1.93	NM_001123653.1
*nod1 (nucleotide-binding oligomerization domain 1)*	F: ACACCAACCTGCACTTCAAGGA R: GAGCGCAGGTGTTTAAAGCTCT	2.07	XM_045718619.1
*nlrc3 (nod-like receptor family CARD domain containing 3)*	F: ACACCAACCTGCACTTCAAGGA R: GAGCGCAGGTGTTTAAAGCTCT	2.05	XM_021565181.2
*nlrc5 (nod-like receptor family CARD domain containing 5)*	F: TGATCGGATCTGTGACTAGTGA R: GGTCTATTCGTGGGCGTTTT	1.9	XM_045688185.1
*nlrx1 (nucleotide-binding oligomerization domain X1)*	F: CGAGCTGCTTTACCTCAACC R: GAAGTGCGCTTTTTGGAGAC	2.01	XM_045724354.1
*saa5 (serum amyloid apolipoprotein, isoform 5)*	F: CGAGCTGCTTTACCTCAACC R: GAAGTGCGCTTTTTGGAGAC	1.99	NM_001146565.1
*trop (troponin)*	F: CGAGCTGCTTTACCTCAACC R: GAAGTGCGCTTTTTGGAGAC	1.99	XM_014145948.2
*muc2 (mucin 2)*	F: CGAGCTGCTTTACCTCAACC R: GAAGTGCGCTTTTTGGAGAC	2.08	XM_045711221.1
*cath2 (cathelicidine 2)*	F: CGAGCTGCTTTACCTCAACC R: GAAGTGCGCTTTTTGGAGAC	1.9	NM_001123573.1

**Table 2:** The highest upregulated genes in Atlantic salmon gills following 7 days of chronic hypoxia.

Day	Acc. Code	Hypoxia (log_2_)	Normoxia (log_2_)	Fold Change	P-Val	FDR P-Val	Description
1	XM_045711221.1	11.38	5.94	43.59	4.55 × 10^−14^	1.19 × 10^−10^	mucin-2-like
	XM_014194811.2	10.81	5.74	33.7	3.46 × 10^−6^	3.00 × 10^−4^	uncharacterized LOC100194720
	XM_014137642.2	12.87	8.6	19.32	2.41 × 10^−27^	1.32 × 10^−22^	cGMP-dependent 3′,5′-cyclic phosphodiesterase
	XM_014154975.2; XM_045700983.1	16.73	12.94	13.77	3.76 × 10^−10^	2.61 × 10^−7^	myelin and lymphocyte protein-like
	NM_001173566.1	12.82	9.37	10.94	2.04 × 10^−23^	3.72 × 10^−19^	cAMP-responsive element modulator
	XM_045717623.1	8.85	5.46	10.45	2.41 × 10^−16^	1.55 × 10^−12^	mucin-2-like
	XM_014198919.2	12.33	8.98	10.19	6.25 × 10^−24^	1.71 × 10^−19^	leucine-rich repeat neuronal protein 4
	XM_014161156.2	12.62	9.33	9.78	1.48 × 10^−6^	2.00 × 10^−4^	polyubiquitin
	XM_014123039.2	13.67	10.44	9.42	1.16 × 10^−18^	1.59 × 10^−14^	mitochondrial basic amino acids transporter-like
	XM_014139302.2	11.74	8.94	6.93	7.30 × 10^−14^	1.67 × 10^−10^	cAMP-specific 3′,5′-cyclic phosphodiesterase 4B
3	XM_014154975.2; XM_045700983.1	16.06	12.68	10.43	7.45 × 10^−10^	1.94 × 10^−6^	myelin and lymphocyte protein-like
	NM_001123573.1	9.83	7.46	5.17	3.99 × 10^−5^	3.20 × 10^−3^	CATH-2 cathelicidin antimicrobial peptide
	XM_014192726.2	10.41	8.13	4.84	2.68 × 10^−5^	0.0025	heme-binding protein 2
	XM_045722586.1	7.19	4.96	4.69	3.35 × 10^−7^	0.0001	kelch-like protein 33
	NM_001141294.1	12.78	10.61	4.5	1.03 × 10^−6^	0.0003	heat shock protein family, member 7 (cardiovascular)
	XM_045704474.1	8	5.85	4.45	2.30 × 10^−10^	7.00 × 10^−7^	kelch-like family member 41a
	XM_014178969.2	6.15	4.01	4.41	2.86 × 10^−5^	2.60 × 10^−3^	gastrin/cholecystokinin type B receptor-like
	NM_001141685.1	8.34	6.35	3.97	1.35 × 10^−8^	1.25 × 10^−5^	SET and MYND domain containing 1b
	XM_045699306.1	6.86	4.89	3.92	1.83 × 10^−6^	0.0004	mitochondrial pyruvate carrier 2-like
	XM_014139803.2	11.52	9.61	3.76	1.67 × 10^−9^	3.16 × 10^−6^	ceramide synthase 2
5	XM_014147274.2	12.16	7.29	29.3	2.00 × 10^−4^	0.0027	eosinophil peroxidase-like
	NM_001146565.1	11.05	7.16	14.8	2.01 × 10^−9^	1.25 × 10^−6^	Serum amyloid A-5
	XM_045708496.1	11.27	7.39	14.79	9.30 × 10^−9^	3.54 × 10^−6^	serum amyloid A-5 protein-like
	XM_014154975.2; XM_045700983.1	15.55	12.12	10.77	1.16 × 10^−7^	1.73 × 10^−5^	myelin and lymphocyte protein-like
	NM_001123573.1	10.1	6.85	9.56	3.62 × 10^−10^	3.81 × 10^−7^	CATH-2 cathelicidin antimicrobial peptide
	XM_014129319.2	8.69	5.68	8.09	4.82 × 10^−8^	9.64 × 10^−6^	uncharacterized LOC106563597
	XM_045711694.1	13.99	11.13	7.27	9.84 × 10^−7^	7.67 × 10^−5^	interferon-induced very large GTPase 1-like
	XM_014166065.2	5.66	2.87	6.93	5.00 × 10^−7^	4.74 × 10^−5^	free fatty acid receptor 2
	XM_014132022.2	14.06	11.54	5.76	1.00 × 10^−4^	0.0019	interferon-induced protein 44-like
	XM_045707633.1	5.81	3.29	5.75	7.59 × 10^−5^	1.50 × 10^−3^	gamma-crystallin M2
7	XM_014143429.2	13.23	9.36	14.61	2.47 × 10^−5^	0.0013	C-C motif chemokine 20
	XM_014167588.2	13.02	9.3	13.2	2.85 × 10^−6^	3.00 × 10^−4^	interferon-induced protein 44-like
	XM_045698191.1	9.03	5.34	12.89	2.00 × 10^−4^	0.0053	nectin-4
	XM_014154975.2; XM_045700983.1	14.94	11.26	12.85	6.25 × 10^−7^	0.0001	myelin and lymphocyte protein-like
	XM_045711300.1	16.04	12.59	10.87	1.39 × 10^−13^	1.85 × 10^−9^	60S ribosomal protein L18a
	NM_001123573.1	10	7.04	7.76	6.87 × 10^−8^	2.32 × 10^−5^	CATH-2 cathelicidin antimicrobial peptide
	XM_014150671.2	7.43	4.74	6.42	9.76 × 10^−9^	5.81 × 10^−6^	uncharacterized LOC106574672
	XM_045699306.1	7.36	4.72	6.21	1.68 × 10^−11^	5.64 × 10^−8^	mitochondrial pyruvate carrier 2-like
	XM_014181918.2	7.92	5.32	6.05	1.00 × 10^−4^	0.0036	caspase-14
	XM_045716357.1	13.19	10.65	5.85	3.20 × 10^−5^	1.60 × 10^−3^	up-regulator of cell proliferation-like

**Table 3:** The highest downregulated genes in Atlantic salmon gills following 7 days of chronic hypoxia.

Day	Acc. Code	Hypoxia (log_2_)	Normoxia (log_2_)	Fold Change	P-Val	FDR P-Val	Description
1	XM_045711445.1	9.16	11.66	−5.63	7.64 × 10^−6^	0.0006	NLR family CARD domain-containing protein 3-like
	XM_045714152.1	9.08	11.51	−5.37	5.37 × 10^−14^	1.28 × 10^−10^	corticosteroid 11-beta-dehydrogenase isozyme 2-like
	NM_001141267.3	9.86	12.25	−5.25	3.27 × 10^−5^	0.0017	ccl21 chemokine (C-C motif) ligand 25a
	XM_045712399.1	8.81	11.08	−4.82	0.0001	0.0046	uncharacterized LOC123733078, transcript variant X1
	XM_045715981.1	12.6	14.71	−4.34	1.51 × 10^−6^	0.0002	NLR family CARD domain-containing protein 3-like
	XM_045701759.1	8.3	10.4	−4.27	4.57 × 10^−9^	2.18 × 10^−6^	G-protein coupled receptor family C group 6 member A-like
	XM_045708598.1	8	10.09	−4.25	1.38 × 10^−7^	3.21 × 10^−5^	uncharacterized LOC123730769
	XM_014134906.2	13.15	15.19	−4.12	8.21 × 10^−9^	3.36 × 10^−6^	intermediate filament protein ON3
	XM_045689853.1	3.62	5.64	−4.05	2.29 × 10^−6^	0.0003	probable global transcription activator SNF2L2
	XM_014198296.2	4.75	6.74	−3.97	1.00 × 10^−11^	1.31 × 10^−8^	coronin-6
3	XM_014143657.2	9.03	13.65	−24.66	2.43 × 10^−9^	0.00000425	proto-oncogene c-Fos
	XM_045711885.1	6.1	9.42	−10.04	1.00 × 10^−4^	6.70 × 10^−3^	NLR family CARD domain-containing protein 3-like
	XM_014206157.2	8.41	11.72	−9.97	1.67 × 10^−12^	3.04 × 10^−8^	proto-oncogene c-Fos-like
	XM_014154479.2	9.71	12.98	−9.67	0.0000124	0.0015	phospholipase A2, group IVF, tandem duplicate 1
	XM_014181789.2	9.74	12.62	−7.37	3.05 × 10^−9^	0.00000463	immediate early response gene 2 protein
	NM_001140121.1	9.41	12.27	−7.28	4.71 × 10^−8^	3.16 × 10^−5^	immediate early response 2
	XM_045706116.1	5.47	7.67	−4.6	8.86 × 10^−6^	1.20 × 10^−3^	semaphorin 7A
	XM_045724365.1	5.53	7.61	−4.23	1.70 × 10^−10^	5.49 × 10^−7^	protein fosB-like
	XM_014142219.2	7.16	9.21	−4.13	1.84 × 10^−8^	0.0000158	extracellular matrix protein 1
	XM_014209872.2	5.34	7.37	−4.1	1.72 × 10^−13^	9.43 × 10^−9^	MIS18 binding protein 1
5	XM_014188155.2	10.72	13.73	−8.08	3.00 × 10^−4^	0.0039	myosin heavy chain, fast skeletal muscle-like
	XM_014158560.2	10.16	12.32	−4.46	1.10 × 10^−3^	9.00 × 10^−3^	ATPase sarcoplasmic/endoplasmic reticulum Ca2+ transporting 1, like
	XM_045710388.1	8.86	10.93	−4.18	2.00 × 10^−4^	0.0025	calpain-2 catalytic subunit-like
	XM_045711775.1	13.03	15	−3.92	0.0000141	0.0005	butyrophilin subfamily 3 member A2-like
	XM_045694719.1	9.05	10.93	−3.7	3.17 × 10^−5^	0.0008	perforin-1-like
	XM_045722067.1	10.81	12.48	−3.18	1.10 × 10^−3^	8.90 × 10^−3^	myosin binding protein C1
	XM_045700092.1	7.03	8.66	−3.1	8.00 × 10^−4^	7.40 × 10^−3^	cytidine monophosphate-N-acetylneuraminic acid hydroxylase-like
	XM_045714152.1	10.08	11.69	−3.06	3.02 × 10^−8^	7.09 × 10^−6^	corticosteroid 11-beta-dehydrogenase isozyme 2-like
	XM_014210475.1	8.26	9.71	−2.73	2.00 × 10^−4^	0.003	actin, alpha cardiac muscle
	XM_014163977.2	14.56	15.99	−2.69	8.84 × 10^−6^	3.00 × 10^−4^	uncharacterized LOC106581733
7	XM_014198541.2	5.99	8.43	−5.43	5.51 × 10^−6^	0.0005	THO complex subunit 3-like
	XM_014150153.2	3.5	5.91	−5.31	1.00 × 10^−4^	4.00 × 10^−3^	carboxypeptidase B2 (plasma)
	XM_045713143.1	3.75	6.05	−4.95	1.74 × 10^−9^	0.00000183	uncharacterized LOC123736104
	XM_045708651.1	11.93	14.19	−4.76	5.46 × 10^−10^	0.000000935	NADH-ubiquinone oxidoreductase chain 4-like
	XM_045708717.1	2.37	4.6	−4.69	1.10 × 10^−9^	0.00000137	lamin-L(III)-like
	XM_045713324.1	8.34	10.57	−4.68	1.73 × 10^−6^	2.00 × 10^−4^	ladderlectin-like
	XM_014135608.2	4.61	6.78	−4.49	2.53 × 10^−10^	5.13 × 10^−7^	serine/threonine-protein kinase 35
	XM_014146915.2	3.68	5.84	−4.45	6.75 × 10^−6^	6.00 × 10^−4^	E3 ubiquitin-protein ligase MARCHF9
	XM_014196010.2	3.2	5.27	−4.22	6.96 × 10^−6^	0.0006	phosphorylase b kinase regulatory subunit alpha, skeletal muscle isoform-like
	XM_014146367.2	2.3	4.35	−4.12	1.53 × 10^−9^	1.78 × 10^−6^	transcription factor HES-5

**Table 4:** The highest upregulated genes in Atlantic salmon gills following 7 days of cyclic hypoxia.

Day	Acc. Code	Hypoxia (log_2_)	Normoxia (log_2_)	Fold Change	P-Val	FDR P-Val	Description
1	XM_014205830.2	8.77	4.49	19.41	3.62 × 10^−6^	0.0013	alpha-1-antitrypsin homolog
	XM_014174610.2	8.57	4.29	19.39	9.80 × 10^−6^	2.20 × 10^−3^	hemopexin a
	XM_014201577.2	8.72	4.68	16.38	1.00 × 10^−4^	0.0089	uncharacterized LOC106605687
	XM_045711221.1	9.78	5.94	14.33	0.00000161	0.0008	mucin-2-like
	XM_014155616.2	8.76	4.98	13.69	5.55 × 10^−7^	0.0004	apolipoprotein A-II
	XM_014156798.2	6.83	3.23	12.12	1.41 × 10^−6^	7.00 × 10^−4^	fibrinogen alpha chain
	XM_045718375.1	8.73	5.86	7.31	8.60 × 10^−6^	2.10 × 10^−3^	complement C3
	XM_014124960.2	4.94	2.36	5.99	2.72 × 10^−5^	4.10 × 10^−3^	zona pellucida sperm-binding protein 3
	XM_045689384.1	7.45	5.1	5.1	3.60 × 10^−5^	0.0048	sodium/potassium/calcium exchanger 5
	XM_045717623.1	7.79	5.46	5.01	1.88 × 10^−9^	9.36 × 10^−6^	mucin-2-like
3	XM_014174610.2	9.05	4.56	22.42	1.10 × 10^−5^	0.0006	hemopexin a
	XM_014156800.2	8.32	3.9	21.52	4.52 × 10^−5^	1.40 × 10^−3^	fibrinogen beta chain-like
	NM_001123663.1	8.92	4.85	16.72	2.00 × 10^−4^	0.004	APA11; apoa1 apolipoprotein A-I
	XM_014201577.2	8.67	4.87	14.02	0.0003	0.0056	uncharacterized LOC106605687
	XM_014205830.2	8.38	4.74	12.51	8.79 × 10^−5^	0.0023	alpha-1-antitrypsin homolog
	XM_014155616.2	8.45	4.9	11.68	4.84 × 10^−8^	1.44 × 10^−5^	apolipoprotein A-II
	XM_014166553.2	8.18	4.77	10.64	1.87 × 10^−5^	8.00 × 10^−4^	fatty acid-binding protein 10-A, liver basic
	XM_014154736.2	6.67	3.36	9.91	4.25 × 10^−12^	1.45 × 10^−8^	ETS domain-containing transcription factor ERF-like
	XM_014125574.2	6.68	3.4	9.76	2.00 × 10^−4^	0.0044	protein AMBP
	XM_014123620.2	7.33	4.26	8.39	1.01 × 10^−5^	5.00 × 10^−4^	alpha-2-HS-glycoprotein 1
5	NM_001123663.1	13.25	5.39	233.08	1.52 × 10^−6^	0.0001	APA11; apoa1 apolipoprotein A-I
	XM_014205830.2	11.76	4.84	120.92	2.93 × 10^−9^	1.87 × 10^−6^	alpha-1-antitrypsin homolog
	XM_014174610.2	12.26	5.39	116.79	5.13 × 10^−8^	0.0000147	hemopexin a
	XM_014201577.2	12.13	5.28	115.38	0.00000152	0.0001	uncharacterized LOC106605687
	XM_014156800.2	11.41	4.92	89.8	3.11 × 10^−7^	0.0000475	fibrinogen beta chain-like
	XM_014189797.2	11.34	5.51	57.14	1.29 × 10^−8^	5.47 × 10^−6^	alpha-2-HS-glycoprotein 2
	XM_014125574.2	9.96	4.6	41.23	1.42 × 10^−7^	2.83 × 10^−5^	protein AMBP
	XM_014123620.2	9.32	4.16	35.81	6.68 × 10^−10^	6.78 × 10^−7^	alpha-2-HS-glycoprotein 1
	XM_014206703.2	10.28	5.24	32.96	2.81 × 10^−7^	0.0000442	serum amyloid P-component-like
	XM_014195266.2	11.65	6.64	32.23	9.02 × 10^−8^	2.10 × 10^−5^	fibrinogen gamma chain
7	XM_045711300.1	16.32	12.59	13.23	8.99 × 10^−15^	4.92 × 10^−11^	60S ribosomal protein L18a
	XM_014147082.1	12.75	9.52	9.32	3.46 × 10^−5^	7.00 × 10^−4^	adhesive plaque matrix protein-like
	XM_014167588.2	12.19	9.3	7.45	9.84 × 10^−6^	0.0003	interferon-induced protein 44-like
	XM_045718799.1	9.65	6.86	6.88	1.2 × 10^−16^	2.19 × 10^−12^	ntl-dependent gene 5
	XM_014163340.2	8.61	5.98	6.2	1.28 × 10^−15^	1 × 10^−11^	paired mesoderm homeobox protein 2A-like
	XM_014124135.2	8.09	5.55	5.8	5.02 × 10^−17^	1.38 × 10^−12^	alanyl (membrane) aminopeptidase a
	XM_045709976.1	6.14	3.61	5.79	8.88 × 10^−12^	1.05 × 10^−8^	uncharacterized LOC106589179
	XM_045719531.1	6.99	4.5	5.61	6.58 × 10^−6^	2.00 × 10^−4^	galectin-3-like
	XM_014209152.2	6.88	4.4	5.57	3.12 × 10^−9^	0.000000697	glycoprotein hormones alpha chain 2-like
	XM_045709679.1	9.07	6.61	5.5	5.85 × 10^−6^	2.00 × 10^−4^	CMRF35-like molecule 5

**Table 5:** The highest downregulated genes in Atlantic salmon gills following 7 days of cyclic hypoxia.

Day	Acc. Code	Hypoxia (log_2_)	Normoxia (log_2_)	Fold Change	P-Val	FDR P-Val	Description
1	XM_045712281.1	12.92	15.06	−4.41	9.44 × 10^−6^	0.0022	uncharacterized LOC106594338
	XM_014186018.2	13.13	15.02	−3.71	7.64 × 10^−5^	7.60 × 10^−3^	stonustoxin subunit beta-like
	XM_014186015.2	12.02	13.78	−3.38	1.16 × 10^−5^	0.0025	uncharacterized LOC106594612
	XM_014203003.2	6.03	7.72	−3.23	0.00000142	0.0007	parvalbumin, thymic CPV3
	XM_014177426.2	4.22	5.91	−3.21	8.50 × 10^−7^	0.0006	family with sequence similarity 131 member Ba
	XM_014185476.2	4.31	5.98	−3.18	1.46 × 10^−6^	8.00 × 10^−4^	regulator of G-protein signaling 7
	XM_045721725.1	6.88	8.41	−2.9	5.15 × 10^−6^	1.50 × 10^−3^	uncharacterized LOC123743752
	XM_014137668.2	9.59	11.12	−2.88	4.94 × 10^−7^	4.00 × 10^−4^	calphotin
	XM_014135827.1	5.28	6.77	−2.8	1.19 × 10^−6^	0.0007	uncharacterized LOC106567027
	XM_014200551.2	3.41	4.87	−2.76	8.75 × 10^−5^	8.20 × 10^−3^	transmembrane protein 222
3	XM_014143657.2	8.74	13.65	−30.09	6.92 × 10^−13^	4.74 × 10^−9^	proto-oncogene c-Fos
	XM_014206157.2	7.54	11.72	−18.18	7.04 × 10^−15^	9.65 × 10^−11^	proto-oncogene c-Fos-like
	XM_014145948.2	4.75	8.44	−12.91	2.72 × 10^−5^	0.001	troponin C, skeletal muscle
	NM_001140121.1	8.67	12.27	−12.12	3.67 × 10^−12^	1.34 × 10^−8^	immediate early response 2
	XM_014130704.2	8.59	11.95	−10.21	2.79 × 10^−16^	1.53 × 10^−11^	transcription factor jun-B-like
	XM_014181789.2	9.3	12.62	−10	2.90 × 10^−13^	2.65 × 10^−9^	immediate early response gene 2 protein
	XM_014203148.2	5.7	8.71	−8.03	1.96 × 10^−15^	5.38 × 10^−11^	immediate early response 2a
	XM_014154479.2	10.15	12.98	−7.13	3.75 × 10^−5^	1.30 × 10^−3^	phospholipase A2, group IVF, tandem duplicate 1
	XM_014188155.2	10.11	12.88	−6.81	1.65 × 10^−5^	0.0007	myosin heavy chain, fast skeletal muscle-like
	NM_001139901.1	11.49	14	−5.7	4.93 × 10^−10^	5.67 × 10^−7^	jun B proto-oncogene
5	XM_045688501.1	6.1	8.04	−3.83	9.19 × 10^−6^	0.0005	cilia- and flagella-associated protein 251-like
	XM_045711775.1	13.44	15	−2.94	2.00 × 10^−4^	4.00 × 10^−3^	butyrophilin subfamily 3 member A2-like
	XM_014167200.2	3.63	5.09	−2.75	4.31 × 10^−6^	0.0003	uncharacterized LOC106583251
	XM_014124587.2	2.58	3.97	−2.63	0.0000216	0.0008	hyaluronidase PH-20-like
	XM_045693490.1	3.1	4.41	−2.49	1.81 × 10^−6^	0.0002	cortexin-3
	XM_014142219.2	8.33	9.52	−2.28	3.00 × 10^−4^	4.80 × 10^−3^	extracellular matrix protein 1
	XM_014132725.2	5.2	6.38	−2.27	9.00 × 10^−5^	2.20 × 10^−3^	Nicalin-1-like
	XM_014190507.2	3.67	4.79	−2.17	2.00 × 10^−4^	3.50 × 10^−3^	homeobox protein engrailed-2b
	XM_014168161.2	2.99	4.09	−2.15	8.90 × 10^−5^	0.0021	E3 ubiquitin-protein ligase RNF182
	XM_014145134.2	2.87	3.96	−2.13	2.00 × 10^−4^	3.20 × 10^−3^	uncharacterized LOC106571733
7	XM_014181954.2	2.99	6.08	−8.48	5.70 × 10^−9^	0.00000114	zinc finger protein 501-like
	XM_045703432.1	6.34	8.87	−5.79	4.86 × 10^−5^	9.00 × 10^−4^	extensin-2-like
	XM_014160917.2	12.44	14.94	−5.63	2.30 × 10^−9^	0.000000545	ras-related and estrogen-regulated growth inhibitor-like protein
	XM_045691455.1	4.37	6.79	−5.35	1.76 × 10^−10^	8.93 × 10^−8^	protein ABHD14A-like
	XM_014198541.2	6.2	8.43	−4.7	3.00 × 10^−4^	0.0031	THO complex subunit 3-like
	XM_014191632.2	2.45	4.51	−4.16	6.00 × 10^−4^	5.50 × 10^−3^	calsequestrin-1-like
	XM_014188541.2	2.83	4.75	−3.78	1.97 × 10^−9^	4.95 × 10^−7^	uncharacterized LOC106597339
	XM_014182434.2	13.49	15.36	−3.65	4.00 × 10^−4^	4.20 × 10^−3^	zymogen granule membrane protein 16
	XM_045711305.1	11.78	13.6	−3.53	5.83 × 10^−7^	0.0000341	dolichyl-diphosphooligosaccharide--protein glycosyltransferase subunit 4
	XM_045711647.1	13.81	15.61	−3.49	8.00 × 10^−4^	7.10 × 10^−3^	zymogen granule membrane protein 16-like

## Data Availability

The data will be made available on request.

## Supplementary Materials

The following supporting information can be downloaded at: https://www.mdpi.com/article/10.3390/ani16132024/s1. Figure S1. Validation of microarray results through RT-qPCR comparison of selected genes.
