# Supplementary material for: Comparative Gill Transcriptomics Reveals Unresolved Inflammation Under Chronic Hypoxia and Molecular Plasticity During Cyclic Hypoxia in Salmo salar
Source: Animals (Basel). 2026 Jul 2;16(13):2024. doi: 10.3390/ani16132024 (PMC13359686; doi:10.3390/ani16132024)
Supplement: Supplementary file 1 [file animals-16-02024-s001.zip › animals-4289792-supplementary.pdf]

Comparative gill transcriptomics reveals unresolved inflammation under chronic hypoxia and molecular plasticity during cyclic hypoxia in *Salmo salar*

Nicolás Salinas-Parra [1‡], Yannick Pombett [1,2‡], Felipe Stambuk [1,2], Matías Ilufi [1], Felipe Ramírez-Cepeda [1], Cristian A. Valenzuela [1], Carlos Soto [3], José Gallardo [4] & Luis Mercado [1*]

‡: These authors contributed equally to this work (NS/YP).

*Corresponding author: luis.mercado@pucv.cl (LM).

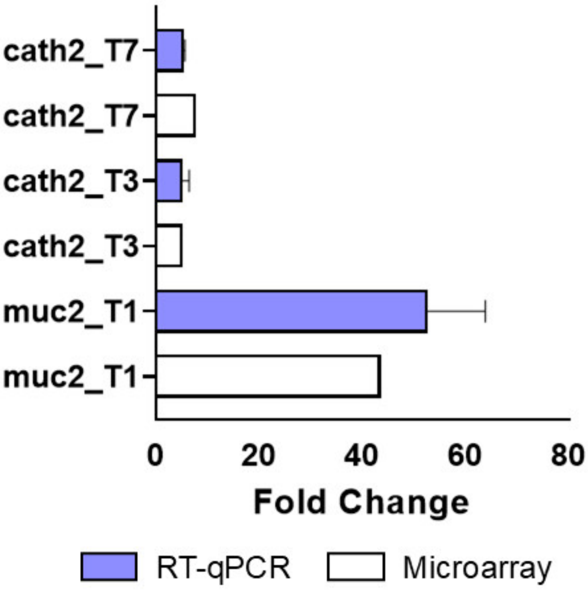

Figure S1. Validation of microarray results through RT-qPCR comparison of selected genes (*cath2* and *muc2*) across three time points: Day 1 (T7), Day 3 (T3), and Day 7 (T7). The data is presented as mean Fold Change ± standard deviation.
